# A non‐interventional study to evaluate the safety and effectiveness of a biphasic, chondrocyte‐containing biomaterial (NOVOCART® 3D) in the treatment of localized full‐thickness cartilage defects or osteochondral lesions of the knee joint (NISANIK)

**DOI:** 10.1002/jeo2.70346

**Published:** 2025-08-13

**Authors:** Julia Elisabeth Lenz, Johannes Weber, Johannes Zellner, Gerald Zimmermann, Peter E. Müller, Alexander Barié, Dominik Popp, Sven Ostermeier, Tilmann Krackhardt, Julian Mehl, Volker Alt, Peter Angele

**Affiliations:** ^1^ Department of Trauma Surgery University Medical Center Regensburg Regensburg Germany; ^2^ Sporthopaedicum Regensburg Straubing Germany; ^3^ Department of Trauma Surgery Theresienkrankenhaus Mannheim Germany; ^4^ Department of Orthopaedics and Trauma Surgery, Musculoskeletal University Center Munich (MUM), University Hospital LMU Munich Munich Germany; ^5^ Department of Sports Orthopaedics and Sports Traumatology University Hospital Heidelberg Heidelberg Germany; ^6^ Center for Joint Surgery and Sport Injuries, Sportopaedie Heidelberg Clinic St. Elisabeth Heidelberg Heidelberg Germany; ^7^ Department of Orthopaedics and Trauma Surgery, Sozialstiftung Bamberg Klinikum am Bruderwald Bamberg Germany; ^8^ UOC Bischberg Bischberg Germany; ^9^ Gelenk‐Klinik Gundelfingen Orthopaedic Clinic Gundelfingen Germany; ^10^ Lubinus Clinicum Kiel Germany; ^11^ Department of Sports Orthopaedics, TUM‐Universitätsklinikum Klinikum rechts der Isar Munich Germany

**Keywords:** cartilage defect, chondrocyte, MACT, matrix‐associated autologous chondrocyte transplantation, osteochondral lesion, transplant

## Abstract

**Purpose:**

The focus of this study was to evaluate the safety and efficacy of NOVOCART® 3D‐treatment over a period of 36 months post‐transplantation.

**Methods:**

This study was designed as a prospective, multicenter, single‐arm, non‐interventional investigation, aimed at evaluating the safety and efficacy of NOVOCART® 3D in patients with localized cartilage defects in the knee joint. 80 patients were enroled across 8 study centres and were followed post‐operatively for a duration of 36 months. Safety assessments were conducted throughout the study period, while effectiveness data were evaluated pre‐operatively and at 3, 12, 18, 24, and 36 months following cell transplantation, utilizing the International Knee Documentation Committee 2000 score (IKDC 2000).

**Results:**

Over the 3‐year observation period among the 80 study patients, the incidence of surgery or product‐related adverse events stood at 12.5%. Subjective scores according to IKDC 2000 demonstrated improvement, with a mean change from baseline of 30.5 ± 21.5 score points at 36 months. Similarly, the mean IKDC function score exhibited continuous enhancement, with a mean difference of 3.2 ± 3.0 score points. These changes from baseline were associated with nominally significant *p*‐values from the 12‐month mark onwards. The subgroup analysis revealed that only higher baseline scores and concurrent surgeries negatively impacted outcome parameters. Female sex, retro‐patellar lesions, uncontained lesions, lesions with intralesional osteophytes or osteochondral defects did not exhibit any significant influence.

**Conclusion:**

The NISANIK study indicates the safety of NOVOCART® 3D treatment. Regarding effectiveness, patients in the study demonstrated a notable and progressively increasing mean improvement compared to their pre‐operative condition. The study furthermore demonstrated that NOVOCART® is universally applicable across all age groups and Body Mass Index ranges, and it can also be effectively used in patients with female sex, larger lesions, retro‐patellar lesions and in such having received bone‐grafting without compromising the outcome, unlike related procedures.

**Level of Evidence:**

Level II, therapeutic, prospective cohort study.

**Abbreviations**
AR: adverse reaction
BMI: body mass index
ICRS: International Cartilage Regeneration and Joint Preservation Society
IKDC: International Knee Documentation Committee
MACT: matrix‐associated autologous chondrocyte transplantation
MMRM: mixed model for repeated measures
OA: osteoarthritis

## INTRODUCTION

Focal cartilage defects of the knee are a risk factor for the development of osteoarthritis. The risk of osteoarthritis increases with the degree and chronicity of the initial cartilage lesion, despite variability in the natural history of chondral lesions [26]. However, there is increasing evidence that adequate repair of such defects can prevent early onset of knee osteoarthritis and delay the need for total knee replacement [10, 19, 35].

The currently available surgical options for the treatment of symptomatic localized, full‐thickness cartilage lesions can be divided into reparative (e.g., bone marrow stimulation) and restorative procedures (e.g., osteochondral transfer and chondrocyte implantation) [2, 3, 11, 31]. Various studies have shown that matrix‐associated autologous chondrocyte transplantation (MACT) formats yield consistently better long‐term patient‐reported functional outcomes and pain levels in comparison to bone marrow stimulation techniques [2, 8, 18, 23, 30, 41]. Furthermore, significantly higher failure rates as well as higher re‐operation rates were shown for microfracture treatment compared to the MACT at long‐term follow‐up [11, 32, 43].

Taken together, the shortcomings of bone marrow stimulation techniques include limited production of hyaline repair tissue, unpredictable repair cartilage volume, frequently observed subchondral bone pathologies, deterioration of results over time, and potential negative impacts on rescue therapies such as cellular transplantation [4, 6, 22, 24, 25]. In addition, the data available suggest that mosaicplasty procedures might be more appropriate for lesions that are smaller than 2–3 cm^2^ and that in this way treated patients are more likely to have inferior outcomes than patients after MACT for a long‐term period [1, 5, 20, 21, 30, 34, 36]. For defects above 3 or 4 cm^2^ in the average sized knee, MACT‐type treatments are the most clinically effective options based on published literature, including Level 1 evidence studies and several meta‐analyses [7, 11, 26, 32, 33, 39].

NOVOCART® 3D is a 3rd generation matrix‐based autologous chondrocyte transplantation system for the treatment of localized full‐thickness cartilage defects of the knee joint. It is composed of in vitro expanded autologous chondrocytes seeded on a bioresorbable biphasic scaffold consisting of a collagen membrane cover attached to a cell‐carrying, three‐dimensional collagen matrix. Previous trials with NOVOCART® 3D have demonstrated promising long‐term results in both adolescents and adults, including high survival rates and sustained functional improvements [9, 12, 27, 28, 37, 38, 42].

The primary study objective was the assessment of safety of tissue harvest and treatment with NOVOCART® 3D by documentation of (serious) adverse events and adverse reactions through 36 months post‐transplantation. The secondary study objective was to assess the improvement of function and performance in adult and adolescent patients treated with NOVOCART® 3D based on clinical data.

## MATERIALS AND METHODS

### Study design

This study was conducted as a prospective, multicenter, single‐arm, non‐interventional study to evaluate the safety and effectiveness of NOVOCART® 3D in patients with localized, full‐thickness cartilage defects in the knee joint. The study was approved by the ‘Paul‐Ehrlich‐Institut’ on 16 April 2015 and the Study No. NIS283 was allocated. Eligible patients were informed and signed an informed consent form prior to participation.

In total, 80 patients were recruited from 8 study centres across Germany between 25 June 2015 and June 2016 (see Table 1). Patients were eligible for participation in this study, if they had a verified diagnosis of localized, clinically symptomatic full‐thickness cartilage defect in the knee joint, caused by acute or repetitive trauma or by osteochondritis dissecans. Recommended treatment indications and contraindications are listed in Table 2.

**Table 1:** Number of enroled patients per study site.

Study site	Number of enroled patients (*n*)
University Clinic, Regensburg	26
Theresienkrankenhaus Mannheim	16
University Clinic, Heidelberg	8
Orthopädische Klinik und Poliklinik, LMU Munich	13
Sozialstiftung, Bamberg	3
Gelenk‐Klinik, Gundelfingen	4
Lubinus Clinicum, Kiel	4
Klinikum rechts der Isar, TU Munich	6
Total	80

**Table 2:** Indications and contraindications for treatment with NOVOCART® 3D.

Indication	Contraindication
Male and female adult patients, or children and adolescents with closed epiphyseal plate	Radiologically apparent osteoarthritis in the target knee as determined by Kellgren and Lawrence grade >2
Defect size ≥2.5 and ≤10 cm^2^. Two defects with a total defect size of up to 20 cm^2^ were treated with two units of NOVOCART® 3D	More than two defects or corresponding lesions
Localized full‐thickness articular cartilage defect of the knee (Grade 3 or 4 according to the International Cartilage Regeneration and Joint Preservation Society [ICRS] classification)	Total/subtotal resected meniscus. Partial resection of up to one‐third of total volume at maximum was considered acceptable
	Diffuse chondromalacia
	Joint stiffness and/or arthrofibrosis
	Insufficient ligament guidance without corrective surgery
	Patella malalignment without correction prior or during ACT
	Inflammatory joint disease (e.g., rheumatoid arthritis)
	Generalized cartilage degeneration or increased wearing of the joint (e.g., osteoarthritis)
	Cancer, present or within the last 5 years
	Primary treatment in children and adolescents with open epiphyseal plate
	Chronic infections (not strictly in patients with hepatitis or HIV)
	Untreated coagulation disorders
	Pregnancy and lactation
	Known history of allergies against ingredients of NOVOCART® 3D

Following the treatment decision for use of NOVOCART® 3D, potentially eligible patients were asked for participation in this study. Consenting patients were then treated according to the NOVOCART® 3D user manual, involving the initial extraction of bone cartilage cylinders for transplant manufacturing at TETEC AG's laboratories, followed by the transplantation of NOVOCART® 3D approximately 3–4 weeks after the initial arthroscopy.

All enroled patients were followed post‐operatively for 36 months. Safety was monitored throughout the study period. Effectiveness data were routinely assessed before the cartilage cell harvesting and 3, 12, 18, 24, and 36 months after cell transplantation.

Study‐specific and standardized paper case report forms were used for systematic documentation of the safety outcomes, joint function and performance following the surgical intervention. The visit schedule planned for each participating subject is provided in Table 3.

**Table 3:** Visit schedule.

Visit number	Type of visit	Time window
1	Screening/baseline	Not applicable
2	Arthroscopy/cell harvesting	Not applicable
3	Transplantation	3–4 weeks post arthroscopy (Visit 2)
4	Hospital discharge	≤7 days post transplantation (Visit 3)
5	3‐month follow‐up	90 ± 14 days post transplantation
6	12‐month follow‐up	365 ± 30 days post transplantation
7	18‐month follow‐up	545 ± 30 days post transplantation
8	24‐month follow‐up	730 ± 30 days post transplantation
9	36‐month follow‐up	1095 ± 30 days post transplantation

### International Knee Documentation Committee (IKDC) 2000 variables

The analysis encompassed the subjective knee evaluation form and the subjective knee form from the IKDC 2000 package.

Considering the IKDC 2000 subjective score, responder rates were calculated, and 95%‐confidence intervals were generated. Response was defined using two different cut‐off scores as an improvement of more than 20.5 score points from baseline, and as an improvement of more than 11.5 score points from baseline. These 2 cut‐off scores were chosen, since Irrgang et al. had suggested that a cut‐off score of 11.5 should be used to maximize the sensitivity of change, whereas a change score of 20.5 should be used to maximize the specificity of change [17].

### Study population

The mean age of the study population was 33.9 ± 11.2 years (15–61 years), 63.8% were males, and 33.8% were current smokers. The median body mass index (BMI) was 26.2 ± 4.7 kg/m^2^ (18.3–38.9) and 21 patients (26.3%) were unable to work because of their knee disorder.

The majority of patients (56.3%) had the left knee affected, and the prevailing causes for the joint cartilage damage were traumatic lesions (37 patients, 46.3%) and osteochondritis dissecans (19 patients, 23.8%). Previous surgical procedures at the target knee were performed in 39 patients (48.8%).

The cartilage defect was mainly located in the femur (59 patients, 73.8%), solely the patella was affected in 18 patients (22.5%), and both the femur and the patella were involved in 3 patients (3.8%). A total of 72 patients (90.0%) had 1 single lesion, and 8 patients (10.0%) had 2 lesions. The mean size of the main lesion (post‐debridement, if available) was 5.1 ± 2.0 cm^2^ and the mean total defect size was 5.4 ± 2.3 cm^2^, ranging from 1.7 to 12.0 cm^2^.

### Surgical procedures required for NOVOCART® 3D treatment

The transplantation of the autologous chondrocytes was performed after a mean interval of 24.4 ± 2.7 days following the initial arthroscopy. In addition, there was one patient with an especially long interval between initial arthroscopy for biopsy harvest and subsequent transplantation of 282 days due to schedule conflicts (not included in the sample statistics).

**Figure 1:**
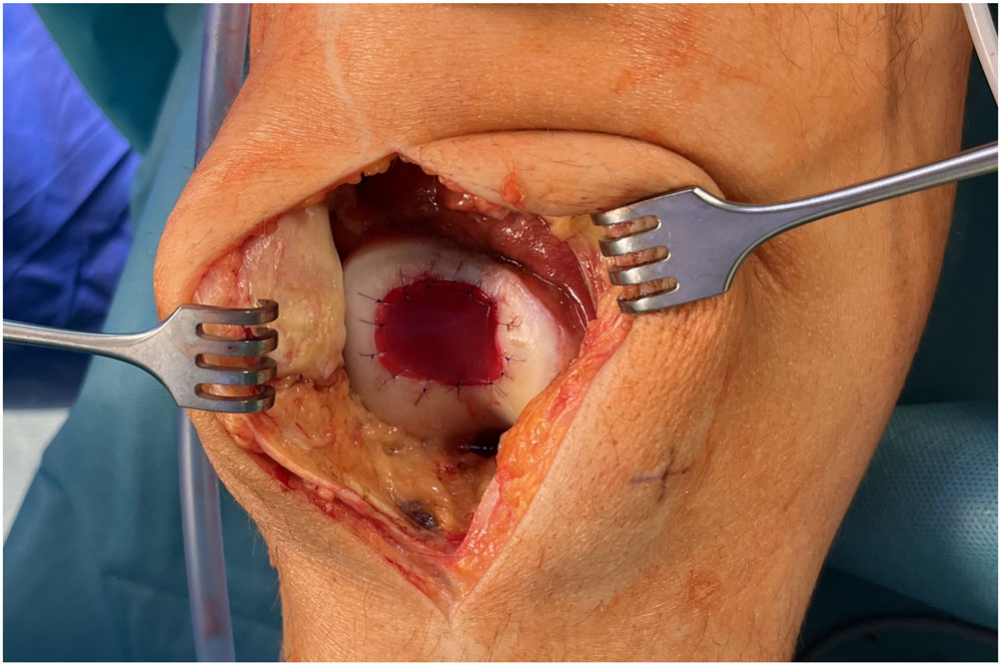
NOVOCART® 3D treatment. Illustrative Example of NOVOCART® 3D Treatment in a Patient with a Chondral Defect of the Right Trochlear Groove.

Transplantation was performed using mini‐arthrotomy. Graft fixation was achieved using sutures alone or in combination with fibrin glue or pins. Postoperatively, patients followed a standardized rehabilitation protocol involving partial weight‐bearing for 6 weeks and continuous passive motion. The transplant was fixed with sutures (Monosyn 6/0) in the majority of patients (60 patients, 75.0%) (see Figure 1). Suture in combination with fibrin glue was used in 12 patients (15.0%) and suture in combination with pins in 5 patients (6.3%). The remaining 3 patients (3.8%) were fixated with fibrin glue only.

The mean duration of hospitalization following the transplantation was 4.3 ± 1.1 days and ranged from 2 to 7 days.

### Concomitantly performed surgical procedures

Additional surgical procedures on the target knee were performed in 71.3% of patients, primarily during the initial arthroscopy or transplantation (see Table 4). Most of the surgeries performed on the day of arthroscopy (47 patients [58.8%] involved) were related to joint cleaning as well as meniscus surgery.

**Table 4:** Concomitant surgeries on the target knee.

Surgery class	Day of arthroscopy, *n* (%)	Day of MACT, *n* (%)	Within 8 weeks after MACT, *n* (%)	Total, *n* (%)
Any surgery	47 (58.8)	41 (51.3)	5 (6.3)	57 (71.3)
Synovectomy	9 (11.3)	17 (21.3)	3 (3.8)	26 (32.5)
Adhesiolysis	17 (21.3)	1 (1.3)	4 (5.0)	19 (23.8)
Bone graft	1 (1.3)	15 (18.8)	1 (1.3)	16 (20.0)
Removal of loose bodies from joint	16 (20.0)	‐	‐	16 (20.0)
Meniscus removal	14 (17.5)	1 (1.3)	‐	15 (18.8)
Ligament operation	1 (1.3)	7 (8.8)	4 (5.0)	12 (15.0)
Joint debridement	5 (6.3)	1 (1.3)	3 (3.8)	9 (11.3)
Meniscus operation	6 (7.5)	1 (1.3)	‐	7 (8.8)
Osteotomy	2 (2.5)	2 (2.5)	3 (3.8)	7 (8.8)
Chondroplasty	1 (1.3)	3 (3.8)	‐	4 (5.0)
Microfracture	0	3 (3.8)	‐	3 (3.8)
Chondroabrasion	1 (1.3)	0	‐	1 (1.3)
Joint dislocation reduction	‐	3 (3.8)	‐	3 (3.8)
Removal of internal fixation	1 (1.3)	1 (1.3)	‐	2 (2.5)
Notch plasty	1 (1.3)	1 (1.3)	‐	1 (1.3)
Bone debridement	‐	1 (1.3)	‐	1 (1.3)
Bursa removal	‐	1 (1.3)	‐	1 (1.3)
Synovial cyst removal	‐	1 (1.3)	‐	1 (1.3)

Abbreviation: MACT, matrix‐associated autologous chondrocyte transplantation.

On the day of transplantation (41 patients [51.3%] involved), the prevailing surgical measures were synovectomy (17 patients, 21.3%), bone grafting (15 patients, 18.8%), and ligament operations (7 patients, 8.8%).

The surgeries electively performed within 8 weeks after transplantation (5 patients [6.3%] involved) included ligament operations (4 patients, 5.0%), osteotomies (3 patients, 3.8%), and various associated joint cleaning measures. These procedures aimed to further optimize biomechanical conditions.

Overall, four patients (5.0%) had concomitantly received chondroplastic treatments in addition to the NOVOCART ® 3D treatment.

### Data management and statistical analysis

Generally, the data of this study were analysed using epidemiological statistical methods. Where appropriate, variability of data was described using 95% confidence intervals. The level of 5% was used to evaluate differences as significant.

Data management and statistical analyses were performed using the software package SAS®, Version 9.2. All study data were entered into a proprietary database application running on the SQL Server 2008‐based infrastructure. The servers are owned by B. Braun and localized in Melsungen, Germany.

Missing values were analysed as such; no imputation methods were applied. If missing values were present in frequency analyses, adjusted relative frequencies were calculated.

Subgroup analyses (based on sex, age, BMI, prior surgeries, concomitant surgeries, number of lesions, lesion size, lesion localization, aetiology, use of analgesics at baseline, and IKDC subjective baseline score), single regression analyses and multiple mixed model for repeated measures (MMRM) analyses were run in order to detect potential differences in the response to treatment with NOVOCART® 3D among the various subgroups and to identify potential response predictors.

## RESULTS

### Follow‐up

The study showed a good follow‐up rate. A total of 13 out of 80 patients (16.3%) had discontinued the study prematurely (see Table 5), resulting in 67 patients (83.8%) who had completed the full study course through 36 months. The number of patients with available visits and IKDC subjective scores/examination grades is provided in Table 6. The mean duration of follow‐up per patient was 32.6 ± 8.6 months and ranged from 0.1 to 40.3 months.

**Table 5:** Disposition of study patients.

	Number of patients (*N* = 80)
Status	*n* (%)
Enroled	80 (100.0)
Arthroscopy and transplantation performed	80 (100.0)
Completed study	67 (83.8)
Discontinued the study prematurely	13 (16.3)
Reason for premature discontinuation^a^	
Subject withdrew consent	2 (15.4)
Subject is lost to follow‐up	11 (84.6)

^a^ Percentages based on the 13 patients with premature termination.

**Table 6:** Disposition of patients.

Type of visit	No. of patients with visit	No. of patients with evaluable IKDC subjective score
Screening/baseline	80	78
Arthroscopy	80	na
Transplantation	80	na
Hospital discharge	80	na
3‐month follow‐up	74	69
12‐month follow‐up	76	76
18‐month follow‐up	60	61
24‐month follow‐up	60	61
36 months follow‐up	66	65

*Note*: Some patients completed their questionnaires at home and sent them by mail to the study sites (i.e., no formal on‐site visit was performed). Therefore, the number of available questionnaires at a given time point might be higher than the number of performed visits.

Abbreviations: IKDC, International Knee Documentation Committee; na, not applicable.

### Safety of NOVOCART® 3D treatment

Adverse reactions (ARs) assessed as related to the NOVOCART® 3D transplant itself or to the surgical procedures (tissue harvesting or transplantation) were reported in 10 out of the 80 study patients (12.5%). All these events represented ‘expected’ clinical complications for ACT treatment. ARs with a suspected relationship to the NOVOCART® 3D product itself were reported in four patients (5.0%), including two patients with transplant failure, two patients with bone marrow oedema, and one patient with graft hypertrophy. A total of eight procedure‐related ARs were seen in seven patients (8.8%) and comprised arthralgia (four patients), intralesional osteophyte (one patient), joint catching (one patient), poor peripheral circulation, meaning transient reduced capillary perfusion in the operated limb without clinical consequences (one patient) and joint effusion (one patient). The re‐operation rate due to ARs was 5% and included one adhesiolysis, one removal of hypertrophic graft tissue and 2 s MACTs.

### IKDC subjective score

**Figure 2:**
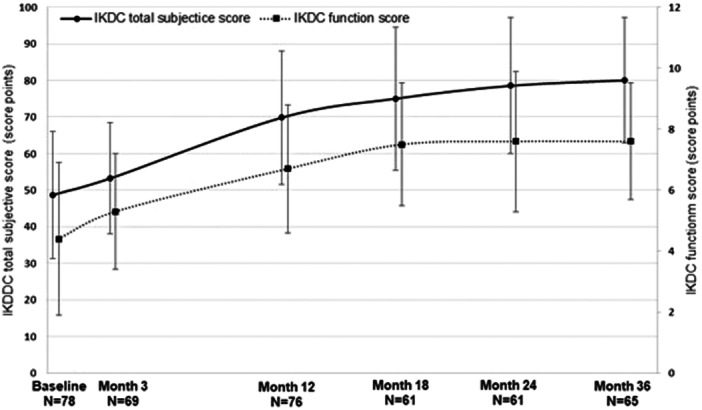
Mean IKDC subjective‐ and function score. Vertical error bars indicate the standard deviations for the respective mean values. IKDC, International Knee Documentation Committee.

The IKDC subjective score was considered the primary effectiveness variable. The mean absolute values over time are displayed in Figure 2. The mean baseline value prior to transplantation of NOVOCART® 3D was 48.7 ± 17.4 score points. Subsequently, the course of the mean subjective score indicated a slight increase at three months (mean change from baseline: 3.5 ± 20.2 score points; mean absolute value: 53.2 ± 15.2 score points) and a stronger improvement at 12 months (mean change from baseline: 20.9 ± 21.7 score points; mean absolute value: 69.8 ± 18.2 score points) with increasing improvement up to 36 months (mean changes from baseline of 27.0 ± 22.4 score points at 24 months and of 30.5 ± 21.5 score points at 36 months; mean absolute values: 78.5 ± 18.6 and 80.0 ± 17.2 score points, respectively).

### IKDC function score

Consistently, the mean IKDC function score increased continuously from 4.4 ± 2.5 score points at baseline to 5.3 ± 1.9 score points at 3 months, 6.7 ± 2.1 score points at 12 months, and 7.6 ± 1.9 score points at 36 months (mean change by 3.2 ± 3.0 score points; see Figure 2).

The changes from baseline were associated with nominally significant *p* value at 12 months onwards (*p* < 0.001 at each time point), and the lower limits of 95% confidence intervals were >0 at all observation time points after 3 months for both subjective and function scores.

### Responder rate

**Figure 3:**
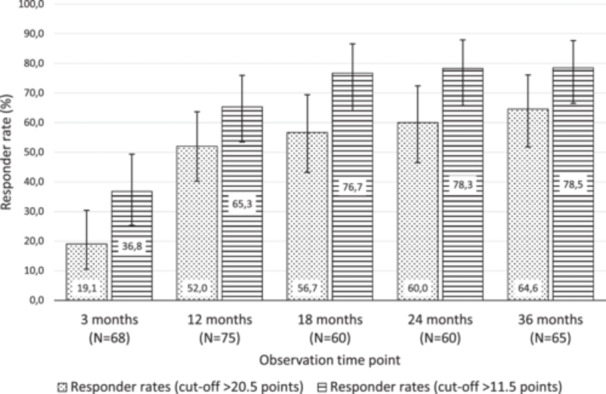
IKDC subjective score—Responder rates by observation time point. Responders were defined as patients who achieved improvement from baseline by 11.5 score points or 20.5 score points, respectively, at a given visit. Vertical error bars indicate 95% confidence intervals for the respective responder rate. IKDC, International Knee Documentation Committee.

In addition, responder rates based on the IKDC subjective score were calculated according to the cut‐off scores proposed by Irrgang and colleagues [17], where a cut‐off score of >11.5 points relative to baseline should be used to maximize the sensitivity of the change, while a change of >20.5 score points from baseline should be used to maximize the specificity of change. Figure 3 displays the responder rates based on these two cut‐off scores for each post‐baseline visit. There was a continuous increase in both responder rates over time; response rates after 3 years were 78.5% based on the more liberal cut‐off score of >11.5 score points and 64.6% based on the stricter cut‐off score of >20.5 score points.

### Subgroup analyses and multivariate regression analyses

Comprehensive subgroup analyses based on sex, age, BMI, prior surgeries, concomitant surgeries, number of lesions, lesion size, lesion localization, aetiology, use of analgesics at baseline, and IKDC subjective baseline score were run in order to detect potential differences in the response to treatment with NOVOCART® 3D among the various subgroups and to identify potential response predictors.

Nominally significant effects at each study visit were observed for the IKDC subjective score at baseline, where poorer baseline conditions were associated with better treatment outcomes. In addition, nominally significant effects were observed for the performance of concomitant surgeries at 12, 24, and 36 months, where patients without concomitant surgeries had better outcomes compared to patients who had undergone such procedures. Nevertheless, clinically relevant and nominally significant improvements from baseline were seen in both subgroups (Table 7). No significant subgroup differences were observed for the other parameters investigated.

**Table 7:** Nominally significant findings (*p* ≤ 0.05) in the analyses for differences in IKDC score across subgroups.

Time point	Subgroup	Patients n (%)	Change from Baseline	95%‐Confidence interval	*p* value
3 months follow‐up	IKDC subjective score at baseline ≤60 points >60 points	49 (72.1) 19 (27.9)	9.4 −11.6	[4.1; 14.7] [−19.5; −3.7]	<0.001
12 months follow‐up	IKDC subjective score at baseline ≤60 points >60 points	54 (72.0) 21 (28.0)	26.9 5.4	[21.4; 32.4] [−2.8; 13.6]	<0.001
	Conc. surgeries on target knee Yes No	54 (72.0) 21 (28.0)	17.5 29.6	[12.1; 22.8] [18.3; 41.0]	0.028
18 months follow‐up	IKDC subjective score at baseline ≤60 points >60 points	43 (71.7) 17 (28.3)	29.8 14.9	[23.4; 36.2] [6.2; 23.6]	0.011
24 months follow‐up	IKDC subjective score at baseline ≤60 points >60 points	41 (68.3) 19 (31.7)	33.5 13.0	[26.8; 40.1] [4.1; 21.9]	<0.001
	Conc. surgeries on target knee Yes No	43 (71.7) 17 (28.3)	22.9 37.3	[16.4; 29.5] [25.8; 48.7]	0.024
36 months follow‐up	IKDC subjective score at baseline ≤60 points >60 points	47 (72.3) 18 (27.7)	36.7 14.4	[30.8; 42.6] [6.5; 22.2]	<0.001
	Conc. surgeries on target knee Yes No	44 (67.7) 21 (32.3)	26.6 38.8	[20.9; 32.2] [27.4; 50.3]	0.030

*Note*: IKDC score at baseline: 48.7 ± 17.4 points. Changes from baseline are given as IKDC score points.

Abbreviation: IKDC, International Knee Documentation Committee.

The regression analyses confirmed the meaning of the baseline IKDC subjective scores for the treatment outcome at each visit but did not indicate any other nominally significant effects of the remaining continuous covariates lesion size, age, and BMI. Likewise, the results of the MMRM supported the impact of the IKDC subjective score at baseline on the treatment outcome at each of the study visits; other nominally significant and consistent effects of potential predictor variables were seen for the performance of prior cartilage repair, where absence of prior cartilage repair was associated with numerically better outcomes compared to patients with prior cartilage repair (significant at 12 months and for the cumulated period from 12 to 36 months).

### Clinical cases

**Figure 4:**
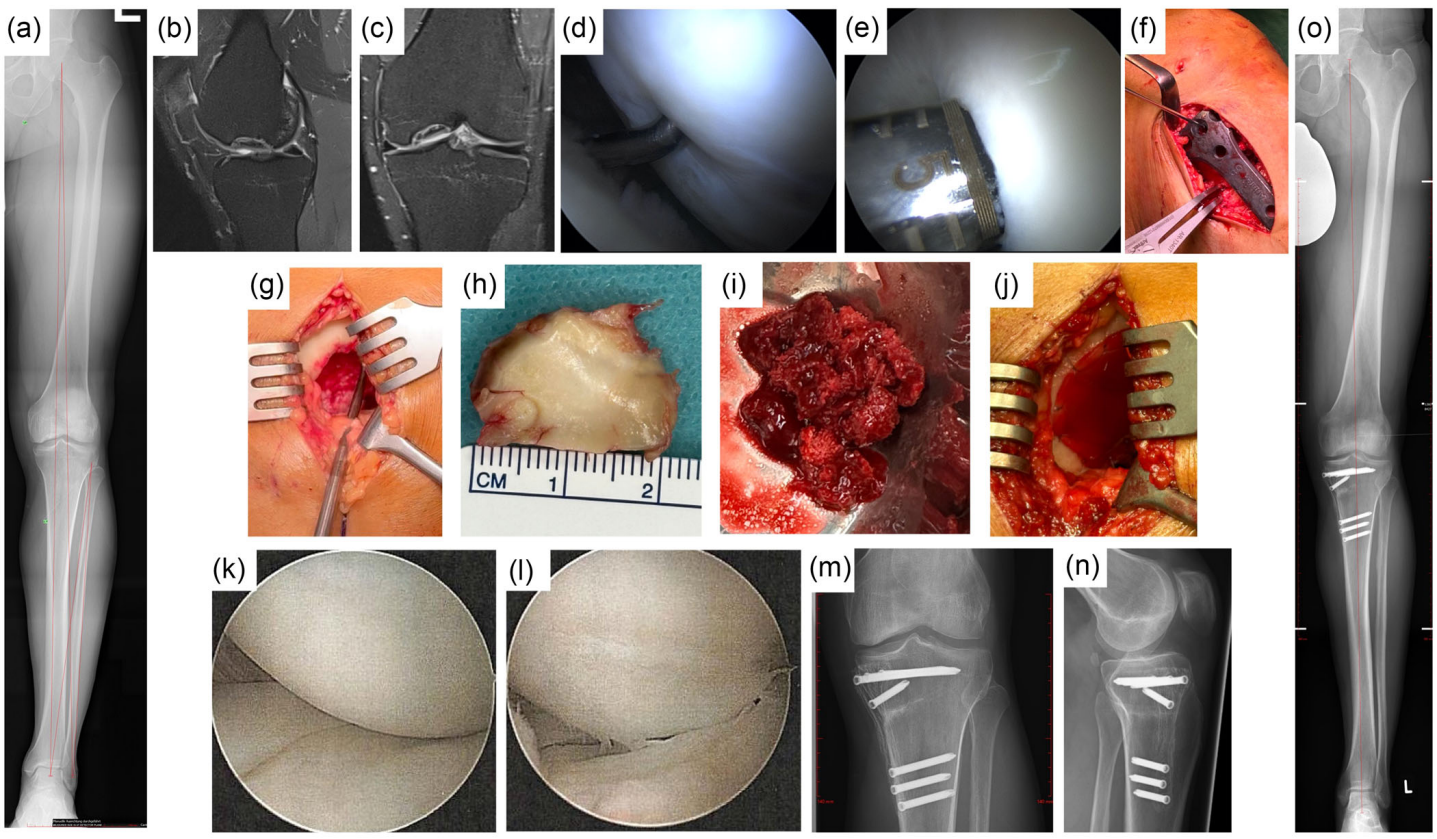
Clinical case No. 1. 34‐year‐old patient with 5° varus deviation (a) and a large and deep OD of the medial femoral condyle (b, c) of the left leg. Arthroscopic view of the OD (d) and chondrocyte harvesting (e) combined with a HTO (f). After 4 weeks the preparation of the defect after removal of the OD (g, h) and osteochondral treatment with cancellous bone grafting (i) combined with a MACT was performed (j). The arthroscopic view of the reconstructed medial condyle by a stable and integrated osteochondral regenerative tissue (k, l) during implant removal after bony healing of the leg axis correction is shown (m, n, o).

**Figure 5:**
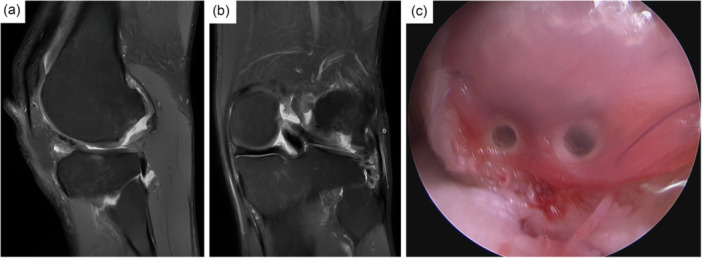
Clinical case No. 2. Different fixation methods available: 37‐year‐old patient with huge osteochondral defect on the lateral femoral condyle. Due to the posterior position, the NOVOCART® 3D implant was fixed besides 6‐0 sutures in the posterior defect aspect with reservable pins (Smart nails, 1,5 mm) after cancellous bone grafting. MRI scans in sagittal (a) and coronal (b) views, along with an arthroscopic view (c), illustrate the fixation approach.

Figures 4 and 5 illustrate two clinical cases successfully treated with NOVOCART® 3D, which are further detailed in the supplementary appendix.

## DISCUSSION

This study was conducted as a prospective, multicenter, single‐arm, non‐interventional study to evaluate the safety and effectiveness of NOVOCART® 3D in patients with localized, full‐thickness cartilage defects in the knee joint.

Adverse reactions (ARs) occurred in 12.5% of patients, with 5% requiring reoperation. All reported ARs represented ‘expected’ clinical complications for (M)ACT treatment. This aligns with findings from Eichinger et al., who reported low complication rates and high patient satisfaction after MACI treatments, with minor revisions required in the mid‐term follow‐up [12]. Similarly, Weishorn et al. noted a long‐term revision‐free survival rate of 97.2% at 10 years, further supporting the safety profile of advanced cartilage repair techniques [38].

The treatment failure rate of 2.5% is consistent with the current literature. Harris and colleagues reported a treatment failure rate with MACTs of 3.3% [16]. Similar results were reported by Wylie et al., with rates for re‐operations of 5% and treatment failures of 3% at 2–5 years follow‐up after MACT [40]. In a systematic review on 93 articles including 3289 patients, Filardo and colleagues reported a 5.2% treatment failure rate with MACT after a mean follow‐up of 34 months [13].

One of the main findings of a recently published study was that NOVOCART® 3D for symptomatic femorotibial and patellofemoral cartilage defects of the knee showed a low graft failure rate of 2.7% and a high survival rate of 97.2% at 9.5 years in a representative cohort of 103 patients (mean patient age: 29.3 years (18–51 years); mean defect size: 4.8 cm^2^ (1.2–12 cm^2^) [38]. In another study by the same group, 27 adolescents and 27 adult patients were followed clinically and radiographically for up to 12 years (mean follow‐up: 96 months) after NOVOCART® 3D treatment of large full‐thickness knee cartilage defects (mean defect size in both groups: 4.6 cm^2^). Osteochondritis dissecans (OCD) was the primary reason for treatment in adolescents, whereas OCD and trauma were the predominant aetiologies in adults. At the time of the last radiographic evaluation none of the patients in the adolescent group showed signs of OA. In the adult control group, one patient showed progression of OA from preoperative Kellgren‐Lawrence grades I to III postoperatively. Three patients had preoperative grade I OA without progression during long‐term follow‐up [37].

In contrast, in their systematic review, Orth et al. reported failure rates of 11%–27% within 5 years after microfracture (mean defect size 3.4 ± 2.1 cm^2^) [29]. A systematic review of level 1 and 2 studies concluded that treatment failure after microfracture can be expected beyond 5 years post‐operatively, regardless of lesion size [15]. This was confirmed in another recent systematic review describing a high rate of OA progression (in 40%–100% of cases) with poor defect healing after microfracture in cartilage defects of 2 to 4 cm^2^ with a mean follow‐up of 10 years and greater [14].

The primary effectiveness measure, the IKDC subjective score, showed a steady and significant improvement over time, increasing from 48.7 ± 17.4 preoperatively to 80.0 ± 17.2 at 36 months. This continuous improvement is comparable to the results of Eichinger et al., who documented significant increases in IKDC scores up to 5–7 years postoperatively [12]. Furthermore, the findings are corroborated by Bumberger et al., who observed substantial improvements in IKDC scores with NOVOCART‐based ACI products over shorter periods [9].

Responder rates at 36 months reached 78.5% based on the >11.5‐point cut‐off and 64.6% based on the stricter >20.5‐point cut‐off. These results are comparable to those reported by Weishorn et al., where a majority of patients achieved clinically meaningful improvements over a similar time frame [37].

In contrast to previous studies, which evaluated NOVOCART® 3D under more controlled conditions, our study provides real‐world data on a broader patient population, including those with comorbidities and concomitant procedures, thereby enhancing generalizability [37, 38].

### Limitations

The generalizability of the analyses separated by subgroups is limited because of the relatively small and heterogeneous sample sizes within certain subgroups. Nevertheless, the nominally significant improvements from baseline in the IKDC subjective score observed in almost all subgroups from 12 months postoperatively and onwards as well as the absence of any robust signs of clinically meaningful differences among subgroups suggest that the treatment with NOVOCART® 3D is similarly beneficial in a broad range of patients with cartilage defects in the knee. This includes patients who were traditionally supposed to benefit less from cartilage repair attempts, such as patients with larger defects, retro‐patellar defects or degenerative cartilage lesions, or patients who had undergone prior cartilage repair procedures on the same defect. Likewise, the subgroup analyses separated by age groups did not indicate relevant differences in the IKDC subjective score changes from baseline, suggesting that treatment with NOVOCART® 3D was effective independently of the patients' age.

An additional limitation is the exclusive use of the IKDC 2000 score as the sole outcome measure. The absence of objective imaging or structural assessments limits the robustness of conclusions regarding treatment effectiveness. In addition, although the presence of previous surgeries was recorded, no detailed information was collected on the type, timing, or extent of these procedures. A further limitation of the present study is the lack of follow‐up imaging, such as MRI. While structural outcome measures like the MOCART score would have provided valuable insights into cartilage repair tissue quality, they were not included due to the non‐interventional nature and associated resource constraints of the study. Moreover, the variability in fixation techniques and concomitant procedures reflects real‐world practice but introduces heterogeneity, which may influence outcomes.

## CONCLUSIONS

The findings from the NISANIK study, spanning a three‐year individual observation period, indicate the safety of NOVOCART® 3D treatment. Regarding effectiveness, patients in the study demonstrated a notable and progressively increasing mean improvement compared to their pre‐operative condition. The study furthermore demonstrated that NOVOCART® 3D is universally applicable across all age groups and BMI ranges, and it can also be effectively used in patients with larger lesions, retro‐patellar lesions and in such having received bone‐grafting without compromising the outcome, unlike related procedures.

## AUTHOR CONTRIBUTIONS

Julia Elisabeth Lenz and Peter Angele drafted the manuscript and performed the analysis. Johannes Weber, Johannes Zellner, and Volker Alt reviewed the manuscript. Gerald Zimmermann, Peter E. Müller, Alexander Barié, Dominik Popp, Sven Ostermeier, Tilmann Krackhardt, Julian Mehl, and Peter Angele performed the operations and collected the study data. All authors read and approved the final manuscript.

## CONFLICT OF INTEREST STATEMENT

P.M. and P.A. consultant/professorship and funded clinical studies of Aesculap/TETEC AG, otherwise no conflicts of interest.

## ETHICS STATEMENT

ClinicalTrials.gov Identifier: NCT02348697. Eligible patients were informed and signed an informed consent form prior to participation. Paul‐Ehrlich‐Institute approval number: NIS283.

## Supporting information

Supporting material.

## Data Availability

The data that support the findings of this study are available from the corresponding author upon reasonable request.

## References

[1] Andrade R , Vasta S , Papalia R , Pereira H , Oliveira JM , Reis RL , et al. Prevalence of articular cartilage lesions and surgical clinical outcomes in football (soccer) players’ knees: a systematic review. Arthroscopy. 2016;32:1466–1477. 27090724 10.1016/j.arthro.2016.01.055

[2] Angele P , Zellner J , Schröter S , Flechtenmacher J , Fritz J , Niemeyer P . Biological reconstruction of localized full‐thickness cartilage defects of the knee: a systematic review of level 1 studies with a minimum follow‐up of 5 years. Cartilage. 2022;13:5–18. 36250517 10.1177/19476035221129571 PMC9924981

[3] Barié A , Kruck P , Sorbi R , Rehnitz C , Oberle D , Walker T , et al. Prospective long‐term follow‐up of autologous chondrocyte implantation with periosteum versus matrix‐associated autologous chondrocyte implantation: a randomized clinical trial. Am J Sports Med. 2020;48:2230–2241. 32667270 10.1177/0363546520928337

[4] Beck A , Murphy DJ , Carey‐Smith R , Wood DJ , Zheng MH . Treatment of articular cartilage defects with microfracture and autologous matrix‐induced chondrogenesis leads to extensive subchondral bone cyst formation in a sheep model. Am J Sports Med. 2016;44:2629–2643. 27436718 10.1177/0363546516652619

[5] Bentley G , Biant LC , Vijayan S , Macmull S , Skinner JA , Carrington RWJ . Minimum ten‐year results of a prospective randomised study of autologous chondrocyte implantation versus mosaicplasty for symptomatic articular cartilage lesions of the knee. J Bone Joint Surg Br. 2012;94–B:504–509. 10.1302/0301-620X.94B4.27495 22434467

[6] Bert JM . Abandoning microfracture of the knee: has the time come? Arthroscopy. 2015;31:501–505. 25744322 10.1016/j.arthro.2014.12.018

[7] Biant LC , McNicholas MJ , Sprowson AP , Spalding T . The surgical management of symptomatic articular cartilage defects of the knee: consensus statements from United Kingdom knee surgeons. Knee. 2015;22:446–449. 26116040 10.1016/j.knee.2015.06.001

[8] Brittberg M , Recker D , Ilgenfritz J , Saris DBF . Matrix‐applied characterized autologous cultured chondrocytes versus microfracture: five‐year follow‐up of a prospective randomized trial. Am J Sports Med. 2018;46:1343–1351. 29565642 10.1177/0363546518756976

[9] Bumberger A , Niemeyer P , Angele P , Wright EK , Faber SO . Hydrogel‐based and spheroid‐based autologous chondrocyte implantation of the knee show similar 2‐year functional outcomes: an analysis based on the German Cartilage Registry (KnorpelRegister DGOU). Knee Surg Sports Traumatol Arthrosc. 2024;32:2258–2266. 38751089 10.1002/ksa.12248

[10] de Windt TS , Vonk LA , Brittberg M , Saris DBF . Treatment and prevention of (Early) osteoarthritis using articular cartilage repair‐fact or fiction? A systematic review. Cartilage. 2013;4:5S–12S. 26069664 10.1177/1947603513486560 PMC4297066

[11] Devitt BM , Bell SW , Webster KE , Feller JA , Whitehead TS . Surgical treatments of cartilage defects of the knee: systematic review of randomised controlled trials. Knee. 2017;24:508–517. 28189406 10.1016/j.knee.2016.12.002

[12] Eichinger M , Henninger B , Petry B , Schuster P , Herbst E , Wagner M , et al. Treatment of cartilage defects in the patellofemoral joint with matrix‐associated autologous chondrocyte implantation effectively improves pain, function, and radiological outcomes after 5‐7 years. Arch Orthop Trauma Surg. 2024;144:1655–1665. 38206448 10.1007/s00402-023-05179-0 PMC10965587

[13] Filardo G , Andriolo L , Balboni F , Marcacci M , Kon E . Cartilage failures. Systematic literature review, critical survey analysis, and definition. Knee Surg Sports Traumatol Arthrosc. 2015;23:3660–3669. 25193571 10.1007/s00167-014-3272-2

[14] Gopinatth V , Jackson GR , Touhey DC , Chahla J , Smith MV , Matava MJ , et al. Microfracture for medium size to large knee chondral defects has limited long‐term efficacy: a systematic review. J Exp Orthop. 2024;11:e70060. 39429888 10.1002/jeo2.70060 PMC11490187

[15] Goyal D , Keyhani S , Lee EH , Hui JHP . Evidence‐based status of microfracture technique: a systematic review of level I and II studies. Arthroscopy. 2013;29:1579–1588. 23992991 10.1016/j.arthro.2013.05.027

[16] Harris JD , Siston RA , Brophy RH , Lattermann C , Carey JL , Flanigan DC . Failures, re‐operations, and complications after autologous chondrocyte implantation—a systematic review. Osteoarthritis Cartilage. 2011;19:779–791. 21333744 10.1016/j.joca.2011.02.010

[17] Irrgang JJ , Anderson AF , Boland AL , Harner CD , Neyret P , Richmond JC , et al. Responsiveness of the International Knee Documentation Committee Subjective Knee Form. Am J Sports Med. 2006;34:1567–1573. 16870824 10.1177/0363546506288855

[18] Jones KJ , Kelley BV , Arshi A , McAllister DR , Fabricant PD . Comparative effectiveness of cartilage repair with respect to the minimal clinically important difference. Am J Sports Med. 2019;47:3284–3293. 31082325 10.1177/0363546518824552

[19] Jungmann PM , Gersing AS , Baumann F , Holwein C , Braun S , Neumann J , et al. Cartilage repair surgery prevents progression of knee degeneration. Knee Surg Sports Traumatol Arthrosc. 2018;27:3001–3013. 30542744 10.1007/s00167-018-5321-8

[20] Li Z , Zhu T , Fan W . Osteochondral autograft transplantation or autologous chondrocyte implantation for large cartilage defects of the knee: a meta‐analysis. Cell Tissue Bank. 2016;17:59–67. 26068598 10.1007/s10561-015-9515-8

[21] Lynch TS , Patel RM , Benedick A , Amin NH , Jones MH , Miniaci A . Systematic review of autogenous osteochondral transplant outcomes. Arthroscopy. 2015;31:746–754. 25617008 10.1016/j.arthro.2014.11.018

[22] Mirza MZ , Swenson RD , Lynch SA . Knee cartilage defect: marrow stimulating techniques. Curr Rev Musculoskelet Med. 2015;8:451–456. 26411978 10.1007/s12178-015-9303-x PMC4630238

[23] Mithoefer K , Della Villa S . Return to sports after articular cartilage repair in the football (soccer) player. Cartilage. 2012;3:57S–62S. 26069609 10.1177/1947603511410419 PMC4297171

[24] Mithoefer K , Venugopal V , Manaqibwala M . Incidence, degree, and clinical effect of subchondral bone overgrowth after microfracture in the knee. Am J Sports Med. 2016;44:2057–2063. 27190069 10.1177/0363546516645514

[25] Moran CJ , Pascual‐Garrido C , Chubinskaya S , Potter HG , Warren RF , Cole BJ , et al. Restoration of articular cartilage. J Bone Jt Surg. 2014;96:336–344. 10.2106/JBJS.L.01329 24553893

[26] Niemeyer P , Albrecht D , Andereya S , Angele P , Ateschrang A , Aurich M , et al. Autologous chondrocyte implantation (ACI) for cartilage defects of the knee: a guideline by the working group “Clinical Tissue Regeneration” of the German Society of Orthopaedics and Trauma (DGOU). Knee. 2016;23:426–435. 26947215 10.1016/j.knee.2016.02.001

[27] Niemeyer P , Angele P , Spiro RC , Kirner A , Gaissmaier C . Comparison of hydrogel‐based autologous chondrocyte implantation versus microfracture: a propensity score matched‐pair analysis. Orthop J Sports Med. 2023;11:23259671231193325. 37655236 10.1177/23259671231193325 PMC10467419

[28] Niethammer TR , Pietschmann MF , Horng A , Roßbach BP , Ficklscherer A , Jansson V , et al. Graft hypertrophy of matrix‐based autologous chondrocyte implantation: a two‐year follow‐up study of NOVOCART 3D implantation in the knee. Knee Surg Sports Traumatol Arthrosc. 2014;22:1329–1336. 23455387 10.1007/s00167-013-2454-7

[29] Orth P , Gao L , Madry H . Microfracture for cartilage repair in the knee: a systematic review of the contemporary literature. Knee Surg Sports Traumatol Arthrosc. 2020;28:670–706. 30659314 10.1007/s00167-019-05359-9

[30] Perdisa F , Filardo G , De Caro F , Andriolo L , Tentoni F , Sessa A , et al. Matrix‐assisted autologous chondrocyte transplantation versus Mosaicplasty: a long‐term comparison. Abstracts of the 13th World Congress of the International Cartilage Repair Society. Naples—Italy, 24–27 September. 2016.

[31] Redondo ML , Naveen NB , Liu JN , Tauro TM , Southworth TM , Cole BJ . Preservation of knee articular cartilage. Sports Med Arthrosc. 2018;26:e23–e30. 30395060 10.1097/JSA.0000000000000226

[32] Riboh JC , Cvetanovich GL , Cole BJ , Yanke AB . Comparative efficacy of cartilage repair procedures in the knee: a network meta‐analysis. Knee Surg Sports Traumatol Arthrosc. 2016;25:3786–3799. 27605128 10.1007/s00167-016-4300-1

[33] Richter DL , Schenck Jr. RC , Wascher DC , Treme G . Knee articular cartilage repair and restoration techniques: a review of the literature. Sports Health. 2016;8:153–160. 26502188 10.1177/1941738115611350 PMC4789925

[34] Robert H . Chondral repair of the knee joint using mosaicplasty. Orthop Traumatol Surg Res. 2011;97:418–429. 21602114 10.1016/j.otsr.2011.04.001

[35] Sanders TL , Pareek A , Obey MR , Johnson NR , Carey JL , Stuart MJ , et al. High rate of osteoarthritis after osteochondritis dissecans fragment excision compared with surgical restoration at a mean 16‐year follow‐up. Am J Sports Med. 2017;45:1799–1805. 28419816 10.1177/0363546517699846

[36] Solheim E , Hegna J , Øyen J , Harlem T , Strand T . Results at 10 to 14 years after osteochondral autografting (mosaicplasty) in articular cartilage defects in the knee. Knee. 2013;20:287–290. 23482060 10.1016/j.knee.2013.01.001

[37] Weishorn J , Wiegand J , Koch KA , Trefzer R , Renkawitz T , Walker T , et al. Favourable clinical outcomes and low revision rate after M‐ACI in adolescents with immature cartilage compared to adult controls: results at 10 years. Knee Surg Sports Traumatol Arthrosc. 2025;33:167–176. 39010715 10.1002/ksa.12359 PMC11716355

[38] Weishorn J , Wiegand J , Zietzschmann S , Koch KA , Rehnitz C , Renkawitz T , et al. Factors influencing long‐term outcomes after matrix‐induced autologous chondrocyte implantation: long‐term results at 10 years. Am J Sports Med. 2024;52:2782–2791. 39276119 10.1177/03635465241270152 PMC11409559

[39] Welch T , Mandelbaum B , Tom M . Autologous chondrocyte implantation: past, present, and future. Sports Med Arthrosc. 2016;24:85–91. 27135292 10.1097/JSA.0000000000000115

[40] Wylie JD , Hartley MK , Kapron AL , Aoki SK , Maak TG . Failures and reoperations after matrix‐assisted cartilage repair of the knee: a systematic review. Arthroscopy. 2016;32:386–392. 26422710 10.1016/j.arthro.2015.07.025

[41] Wylie JD , Hartley MK , Kapron AL , Aoki SK , Maak TG . What is the effect of matrices on cartilage repair? A systematic review. Clin Orthop Relat Res. 2015;473:1673–1682. 25604876 10.1007/s11999-015-4141-0 PMC4385356

[42] Zak L , Albrecht C , Wondrasch B , Widhalm H , Vekszler G , Trattnig S , et al. Results 2 years after matrix‐associated autologous chondrocyte transplantation using the Novocart 3D scaffold: an analysis of clinical and radiological data. Am J Sports Med. 2014;42:1618–1627. 24817007 10.1177/0363546514532337

[43] Zamborsky R , Danisovic L . Surgical techniques for knee cartilage repair: an updated large‐scale systematic review and network meta‐analysis of randomized controlled trials. Arthroscopy. 2020;36:845–858. 32139062 10.1016/j.arthro.2019.11.096

