# Supplementary material for: A non‐interventional study to evaluate the safety and effectiveness of a biphasic, chondrocyte‐containing biomaterial (NOVOCART® 3D) in the treatment of localized full‐thickness cartilage defects or osteochondral lesions of the knee joint (NISANIK)
Source: J Exp Orthop. 2025 Aug 13;12(3):e70346. doi: 10.1002/jeo2.70346 (PMC12344510; doi:10.1002/jeo2.70346)
Supplement: Supplementary file 1 — Supporting material. [file JEO2-12-e70346-s001.docx]

**Supplementary appendix**

Clinical cases

Figures 4 and 5 present two clinical cases that were successfully performed using NOVOCART^®^3D.

Figure 4 illustrates the case of a 34-year-old patient with a 5° varus deviation and a large, deep osteochondral defect (OD) of the medial femoral condyle in the left leg. Arthroscopic visualization of the OD and chondrocyte harvesting were performed in combination with a high tibial osteotomy (HTO). After four weeks, the defect was prepared following the removal of the OD, and osteochondral treatment was carried out using cancellous bone grafting combined with matrix-assisted autologous chondrocyte transplantation (MACT). Arthroscopic examination during implant removal after bony healing of the leg axis correction confirmed a stable and integrated osteochondral regenerative tissue in the reconstructed medial condyle.

Figure 5 illustrates the case of a 37-year-old patient with a large osteochondral defect on the lateral femoral condyle. Due to the posterior location of the defect, standard suture fixation alone was not sufficient to ensure implant stability. Therefore, after cancellous bone grafting, the NOVOCART® 3D implant was fixed using both 6-0 sutures and resorbable pins (Smart Nails, 1.5 mm) in the posterior aspect of the defect. MRI images in sagittal and coronal views, along with an arthroscopic view, demonstrate the precise localization of the defect and the applied fixation technique. This case highlights the versatility of fixation methods required to adapt to challenging anatomical positions.
